# Ultrasound-Guided Mandibular Alveolar Nerve Block in Rabbits: A Cadaveric Comparison of In-Plane and Out-of-Plane Approaches

**DOI:** 10.3390/vetsci13020135

**Published:** 2026-01-29

**Authors:** Matteo Serpieri, Andrea Degiovanni, Giuseppe Bonaffini, Elena Passarino, Giuseppe Quaranta, Mitzy Mauthe von Degerfeld

**Affiliations:** 1 Department of Veterinary Sciences, University of Turin, Largo Braccini 2, 10095 Grugliasco, Italy; matteo.serpieri@unito.it (M.S.); andrea.degiovanni@unito.it (A.D.); elena.passarino@unito.it (E.P.); giuseppe.quaranta@unito.it (G.Q.); mitzy.mauthe@unito.it (M.M.v.D.); 2 Department of Veterinary Sciences, University of Messina, 98168 Messina, Italy

**Keywords:** cadaveric study, dental disease, locoregional analgesia, mandibular alveolar nerve block, rabbit, ultrasound-guided regional anaesthesia

## Abstract

Mandibular dental disease is common in pet rabbits and often requires invasive procedures associated with significant nociceptive input. Mandibular alveolar nerve blocks have been described in this species using blind techniques; however, their accuracy has not been objectively evaluated, and ultrasound-guided approaches have not been reported. This cadaveric study aimed to describe an ultrasound-guided mandibular alveolar nerve block in rabbits and to compare in-plane and out-of-plane approaches. Twelve adult New Zealand White rabbit cadavers (24 mandibular alveolar nerves) were included. For each rabbit, one nerve was assigned to an in-plane ultrasound-guided approach, while the contralateral nerve was assigned to an out-of-plane approach. Following ultrasound-guided needle placement, 0.1 mL/kg of a mixture of 2% lidocaine and 1% methylene blue was injected. Ultrasound image quality and perineural staining were assessed and scored, and longitudinal dye spread was measured after anatomical dissection. Both approaches allowed consistent identification of the target region and resulted in comparable ultrasound image quality scores, staining scores, and longitudinal dye spread, with no statistically significant differences between techniques. These results demonstrate the anatomical feasibility of ultrasound-guided mandibular alveolar nerve blocks in rabbits using either approach. Further in vivo studies are required to assess clinical efficacy and safety.

## Simple Summary

Dental procedures involving the lower jaw are common in pet rabbits and are often associated with significant pain. Effective perioperative analgesia is therefore essential, particularly in a species that may show subtle signs of pain and is highly sensitive to stress. The mandibular alveolar nerve block can provide targeted analgesia for procedures involving the mandibular teeth; however, in rabbits, it has been described only as a blind technique based on anatomical landmarks. In this cadaveric study, we describe an ultrasound-guided approach to the mandibular alveolar nerve in rabbits and compare two different needle insertion techniques. Ultrasound guidance allowed visualisation of relevant anatomical structures and needle placement near the target nerve. Both techniques resulted in a comparable distribution of the injected solution around the nerve, suggesting that either approach may be technically suitable. The use of an ultrasound-guided approach may be particularly useful in challenging cases or in animals with altered anatomy. Further clinical studies in live rabbits are now needed to confirm its effectiveness and safety in routine practice.

## 1. Introduction

Effective perioperative analgesia is a cornerstone of modern veterinary medicine and represents a fundamental component of welfare-oriented clinical practice. In companion animals, the use of multimodal analgesic protocols combining systemic and locoregional techniques is widely recommended to improve analgesic efficacy, reduce anaesthetic requirements, and minimise perioperative morbidity [1,2]. In recent years, locoregional anaesthesia has gained increasing attention across veterinary species, including small companion mammals, as a valuable adjunct to general anaesthesia and systemic analgesia [3,4,5,6]. Nevertheless, the application of locoregional techniques in rabbits and exotic species remains limited by anatomical variability, small patient size, and the relative scarcity of species-specific, evidence-based guidelines [1]. Among these species, rabbits (*Oryctolagus cuniculus*) are particularly susceptible to pain-related complications due to their prey nature, specific behavioural expression of pain, and high sensitivity to stress and anaesthetic drugs [7,8]. Dental and oromaxillofacial disorders are among the most common clinical conditions encountered in pet rabbits, with odontogenic disease representing a major cause of pain, anorexia, and reduced quality of life [9,10,11]. Surgical and dental procedures involving the mandible and mandibular cheek teeth are therefore frequent in clinical practice and are often associated with significant nociceptive input, making adequate perioperative analgesia essential [12,13,14]. Consequently, the integration of locoregional techniques into analgesic protocols for oral and maxillofacial procedures in rabbits has been increasingly advocated [3,15]. In this context, effective regional anaesthesia of the mandibular alveolar (inferior alveolar) nerve is particularly desirable to provide intraoperative analgesia and to minimise perioperative stress in a species known for its sensitivity to pain and handling [15].

The mandibular alveolar nerve, a branch of the mandibular division of the trigeminal nerve, provides sensory innervation to the mandibular teeth, alveolar bone, gingiva, and lower lip [15,16]. Accordingly, blockade of this nerve is of particular clinical relevance for mandibular dental procedures, as it can provide intraoperative analgesia and contribute to postoperative pain control. In animals and humans, inferior alveolar nerve blocks are routinely performed and have been investigated using blind, neurostimulator-guided, and ultrasound-guided approaches [17,18,19,20,21,22,23,24]. In rabbits, mandibular alveolar nerve blocks have been described exclusively as blind techniques, relying on external anatomical landmarks and estimated distances to the mandibular foramen [15,25]. Jekl & Hauptman [15] provided a detailed description of the blind approach in rabbits, highlighting both its potential clinical utility and its inherent limitations related to anatomical variability and the lack of direct confirmation of needle placement. Blind techniques inherently rely on anatomical assumptions and operator experience, and their actual accuracy, safety, and effectiveness in rabbits have not been objectively evaluated through cadaveric or clinical studies. Notably, no studies have quantified perineural injectate distribution following blind mandibular alveolar nerve blocks in this species.

Moreover, across veterinary species, blind inferior alveolar nerve blocks have been associated with inconsistent success rates and potential complications, including nerve trauma and block failure [17,18,19]. In response to these limitations, ultrasound guidance has been increasingly adopted, allowing real-time visualisation of anatomical landmarks, needle trajectory, and injectate spread, thereby improving accuracy and consistency [21,26]. Despite these advances, ultrasound-guided mandibular alveolar nerve blocks have not yet been described or evaluated in rabbits.

Given the clinical relevance of mandibular dental disease in rabbits, their documented susceptibility to pain, and the limitations of currently described blind techniques, there is a need to develop an ultrasound-guided approach to the mandibular alveolar nerve in this species. The aim of the present cadaveric study was therefore to describe an ultrasound-guided mandibular alveolar nerve block technique in rabbits and to compare in-plane and out-of-plane approaches by assessing ultrasound image quality and perineural dye distribution.

## 2. Materials and Methods

### 2.1. Animals

Twelve adult New Zealand White rabbit cadavers, approximately 3–4 years of age, providing a total of 24 mandibular alveolar nerves, were included in the study. The sample size was determined based on previously published cadaveric studies investigating ultrasound-guided locoregional nerve blocks in rabbits [27,28,29,30,31].

All subjects were cadavers of rabbits that had been humanely euthanised for reasons unrelated to the present study and subsequently donated to the Veterinary Teaching Hospital of the University of Turin for educational and research purposes, with informed owner consent. As all procedures were performed exclusively on cadaveric specimens, ethical committee approval was not required.

Cadavers were stored under refrigeration (0–4 °C) following euthanasia, and all procedures were performed within three days. Prior to the procedures, cadavers were allowed to reach room temperature. All heads were inspected to exclude gross anatomical abnormalities affecting the mandibular region.

### 2.2. Study Design and Mandibular Alveolar Nerve Block Procedure

For each rabbit, one mandibular alveolar nerve (*nervus alveolaris mandibularis*) was randomly allocated (www.randomizer.org; accessed on 10 September 2025) to an in-plane ultrasound-guided approach, while the contralateral nerve was assigned to an out-of-plane approach. All ultrasound-guided procedures and injections were performed by the same operator (M.S., experienced in ultrasound-guided nerve block techniques in rabbits) to minimise inter-operator variability.

Each cadaver was positioned in dorsal recumbency, with the head oriented towards the operator. The intermandibular and cervical regions were clipped to allow adequate ultrasound probe contact and image acquisition. Alcohol was applied to the skin to improve acoustic coupling.

Ultrasound examinations were performed using an ultrasound machine (Philips EPIQ Elite, Philips S.p.A., Milan, Italy) equipped with a high frequency linear array transducer (Philips eL 18-4, Philips S.p.A., Milan, Italy). The probe footprint, defined as the transducer surface in contact with the skin, measured approximately 6.0 cm × 1.5 cm, while the acoustic lens (active imaging area) measured approximately 5.0 cm × 1.0 cm, as determined by direct measurement.

**Figure 1:**
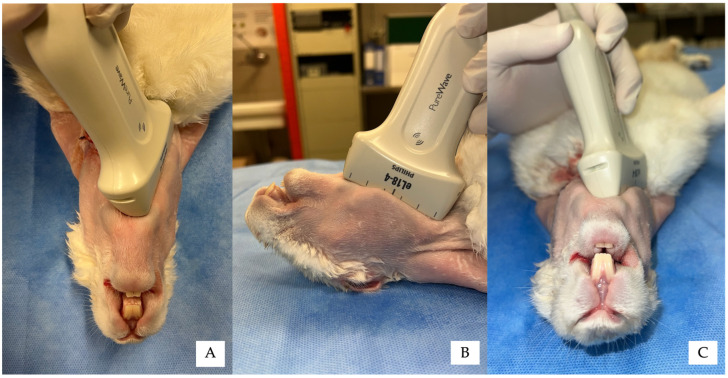
Positioning of the ultrasound transducer for the ultrasound-guided mandibular alveolar nerve block in a rabbit cadaver, in dorsal recumbency. The transducer is placed immediately medial to the mandibular body, with the probe marker oriented caudally. The transducer follows the profile of the mandibular body, allowing visualisation of the medial mandibular surface. (**A**) Ventral view. (**B**) Lateral view. (**C**) Frontal view.

For both approaches, the transducer was positioned immediately medial to the mandibular body (*corpus mandibulae*), with one end placed just caudal to the mandibular symphysis and the opposite end caudomedial to the mandibular angle, following the profile of the mandibular body (Figure 1). The probe marker was oriented caudally.

**Figure 2:**
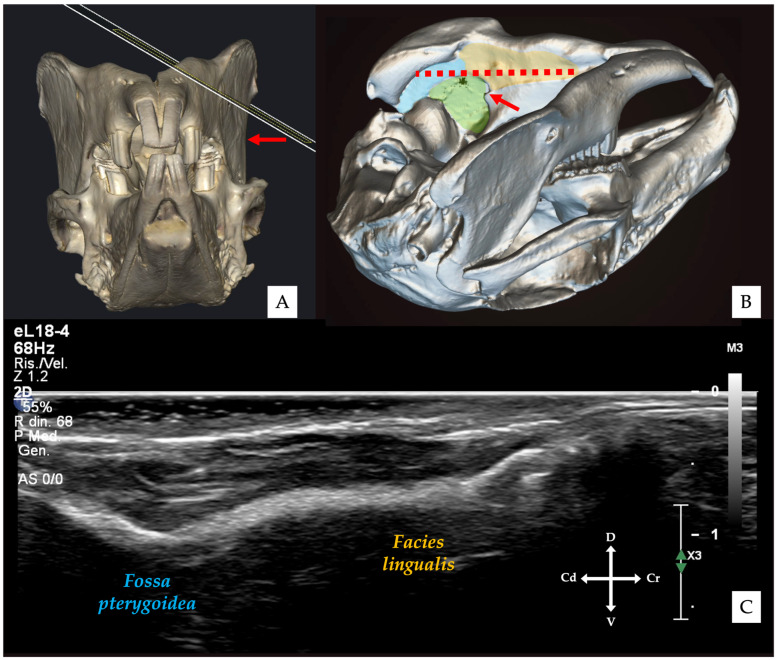
Three-dimensional reconstructions of the rabbit skull illustrating transducer orientation and target anatomical landmarks. (**A**) Frontal view showing the orientation of the ultrasound beam relative to the mandibular body; the red arrow indicates the external surface point corresponding to the mandibular foramen. (**B**) Oblique lateral view highlighting the relationship between the medial surface of the mandibular body, including the *facies lingualis* (yellow area), the *fovea pterygoidea* (green area), and the *fossa pterygoidea* (blue area). The red arrow indicates the mandibular foramen, while the dotted red line represents the ultrasound scanning plane. (**C**) Corresponding ultrasound image obtained with the transducer positioned as shown, illustrating the medial bony profile of the mandibular body at the level of the *facies lingualis* and *fossa pterygoidea*.

**Figure 3:**
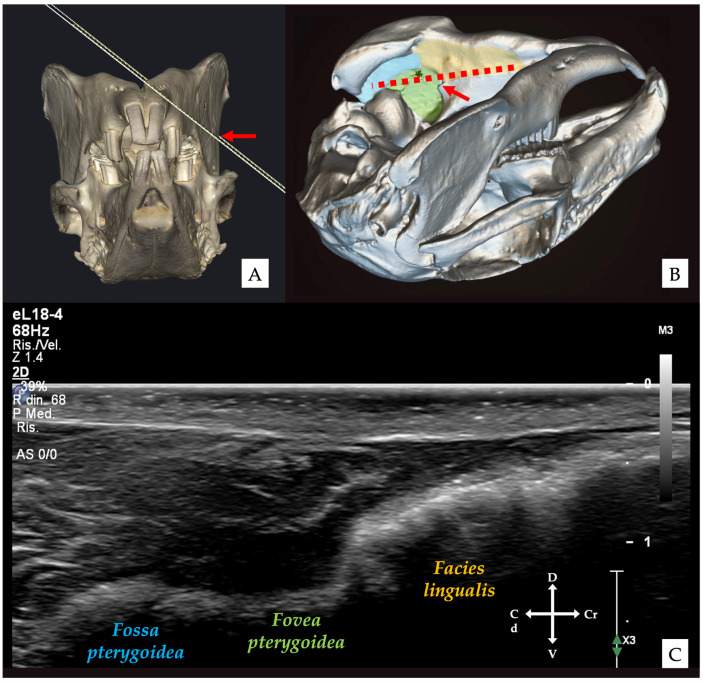
Three-dimensional reconstructions of the rabbit skull illustrating transducer orientation and target anatomical landmarks, for the visualisation of the *fovea pterygoidea*. (**A**) Frontal view showing the orientation of the ultrasound beam relative to the mandibular body; the red arrow indicates the external surface point corresponding to the mandibular foramen. (**B**) Oblique lateral view highlighting the relationship between the medial surface of the mandibular body, including the *facies lingualis* (yellow area), the *fovea pterygoidea* (green area), and the *fossa pterygoidea* (blue area). The red arrow indicates the mandibular foramen, while the dotted red line represents the ultrasound scanning plane. (**C**) Corresponding ultrasound image obtained with the transducer positioned as shown, illustrating the medial bony profile of the mandibular body at the level of the *facies lingualis* and *fossa pterygoidea*, with the ventral aspect of the *fovea pterygoidea* visible.

The transducer was initially angled approximately 30–45° relative to the mandibular body to visualise the medial mandibular surface. In this projection, the cortical profile appeared smooth and continuous, corresponding to the *facies lingualis* surface of the mandibular body, continuing dorsally into the *fossa pterygoidea* (Figure 2 and Figure 3) [32,33]. Subsequently, a “fanning” manoeuvre [34] was performed, increasing the angle to approximately 45–60°, allowing visualisation of a slightly more dorsal portion of the medial mandibular surface. At this level, the ventral aspect of the *fovea pterygoidea* became apparent; its cranial portion corresponded to the region of the mandibular foramen (Figure 2B). Once identified, the *fovea pterygoidea* was centred within the ultrasound image.

**Figure 4:**
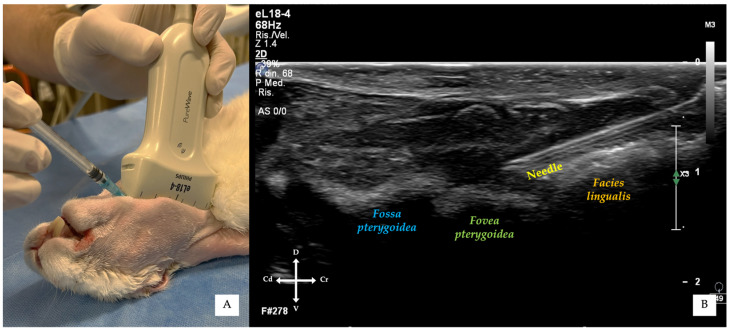
In-plane ultrasound-guided mandibular alveolar nerve block. (**A**) Ventrolateral external view showing transducer positioning and needle insertion just caudal to the mandibular symphysis. (**B**) Corresponding ultrasound image obtained during the procedures, demonstrating continuous visualisation of the needle shaft advancing towards the target area adjacent to the cranial portion of the *fovea pterygoidea*.

**Figure 5:**
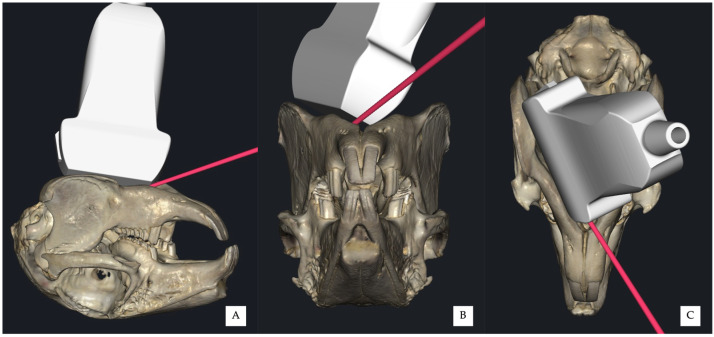
Three-dimensional schematic representations illustrating the in-plane approach for the ultrasound-guided mandibular alveolar nerve block. The red line indicates the needle trajectory. (**A**) Lateral view, (**B**) frontal view, and (**C**) ventral view showing the spatial relationship between the ultrasound transducer, needle trajectory, and the mandibular body.

For the in-plane approach, a 24G hypodermic needle (BD Microlance 3, 24G × 1–1/4”–0.6 mm × 30 mm, BD Italia, Milan, Italy) connected to a syringe was advanced under continuous ultrasound visualisation from a point just caudal to the mandibular symphysis, at an angle of approximately 30° relative to the transducer, until the needle tip was positioned adjacent to the cranial portion of the *fovea pterygoidea*, without touching the bony surface (Figure 4 and Figure 5).

**Figure 6:**
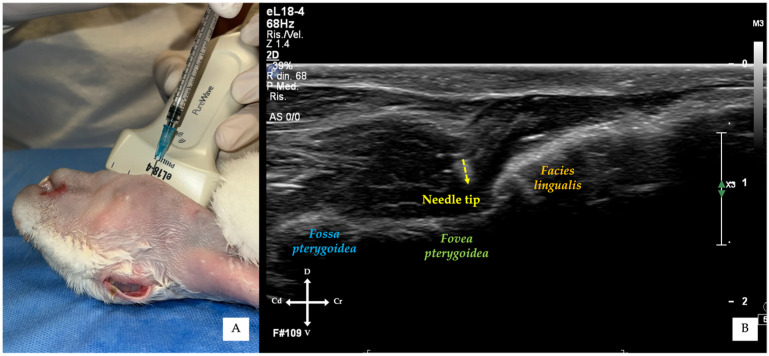
Out-of-plane ultrasound-guided mandibular alveolar nerve block. (**A**) Lateral external view showing transducer positioning and needle insertion immediately medial to the mandibular body, between the transducer and the mandible, at the level of the central probe marker. Notably, in the out-of-plane approach, the needle insertion point is slightly caudal to the mandibular notch. (**B**) Corresponding ultrasound image obtained during the procedures, demonstrating visualisation of the needle tip at the level of the cranial portion of the *fovea pterygoidea*.

**Figure 7:**
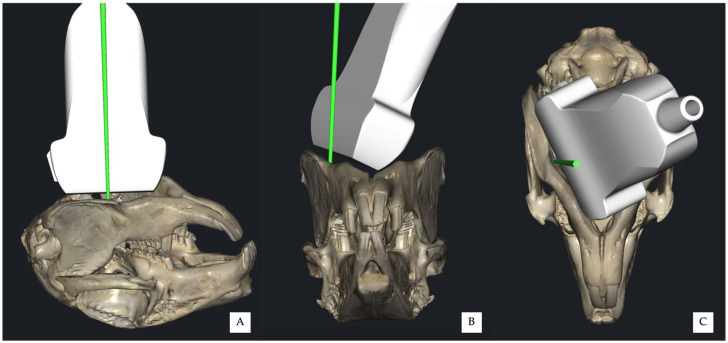
Three-dimensional schematic representations illustrating the out-of-plane approach for the ultrasound-guided mandibular alveolar nerve block. The green line indicates the needle trajectory. (**A**) Lateral view, (**B**) frontal view, and (**C**) ventral view showing transducer positioning and needle trajectory.

For the out-of-plane approach, the needle was inserted immediately medial to the mandible, between the mandibular body and the transducer, entering at the central probe marker (Figure 6 and Figure 7). Correct needle placement was confirmed by visualisation of the needle tip at the level of the *fovea pterygoidea*, without touching the bony surface.

Once the needle tip was correctly positioned, 0.1 mL/kg of a mixture of 2% lidocaine hydrochloride (Lidocaina 20 mg/mL, Ecuphar Italia S.r.l., Milan, Italy) and 1% methylene blue (Blu di Metilene S.A.L.F. 1%, SALF S.p.A., Cenate Sotto, BG, Italy) was injected under ultrasound guidance.

### 2.3. Dissection Procedure and Assessment of Nerve Staining

Following completion of the injection procedures, all cadavers underwent anatomical dissection to evaluate dye distribution.

The skin of the intermandibular region was incised and reflected, and the submandibular salivary glands were removed. For each hemimandible, the digastric muscle was transected, followed by sectioning and reflection of the cranial portion of the pterygoid muscle, allowing exposure of the mandibular alveolar nerve at its entry into the mandibular foramen. The nerve was then carefully dissected free from surrounding tissues to follow its course as far as anatomically possible.

All dissections and evaluations of dye distribution were performed in a blinded manner by a second operator (G.B.), unaware of the ultrasound-guided approach used.

The longitudinal extent of methylene blue staining along the nerve was measured (mm) using a calliper. The presence or absence of circumferential staining was assessed and incorporated into the overall staining score (Section 2.4).

### 2.4. Scoring System

For each mandibular alveolar nerve block, ultrasound image quality was assessed using a semi-quantitative scoring system adapted from Mirra et al. [35]. Three criteria were evaluated:

- clear visualisation of the *fovea pterygoidea*;
- visualisation of the needle (entire shaft for the in-plane approach or needle tip for the out-of-plane approach);
- ultrasound visualisation of injectate spread during injection.

A score of 0 (poor), 1 (good), or 2 (excellent) was assigned when one or none, two, or all three criteria were fulfilled, respectively.

A separate scoring system was used to evaluate the accuracy of perineural injectate deposition [36,37,38] as follows:

- 0 (poor): no staining or longitudinal staining < 6 mm;
- 1 (good): longitudinal staining ≥ 6 mm without circumferential involvement;
- 2 (excellent): longitudinal staining ≥ 6 mm with circumferential staining of the nerve.

### 2.5. Statistical Analysis

Statistical analyses were performed using R software (Version 4.4.3., R Foundation for Statistical Computing, Vienna, Austria). Comparisons between approaches were conducted using paired statistical tests. Normality of paired differences for continuous variables was assessed using the Shapiro–Wilk test. Ordinal variables (ultrasound image quality and staining scores) and non-normally distributed continuous variables (longitudinal staining length, mm) were compared using the Wilcoxon signed-rank test. When paired differences in longitudinal staining length met assumptions of normality, a paired *t*-test was applied. Statistical significance was set at *p* < 0.05.

## 3. Results

Continuous variables are reported as the median (interquartile range), while ordinal and categorical variables are reported as the number (percentage).

The median body weight of the rabbits was 3.0 kg (2.8–3.1 kg). Results for longitudinal nerve staining are summarised in Table 1, while ultrasound image quality scores and staining scores are reported in Table 2.

**Figure 8:**
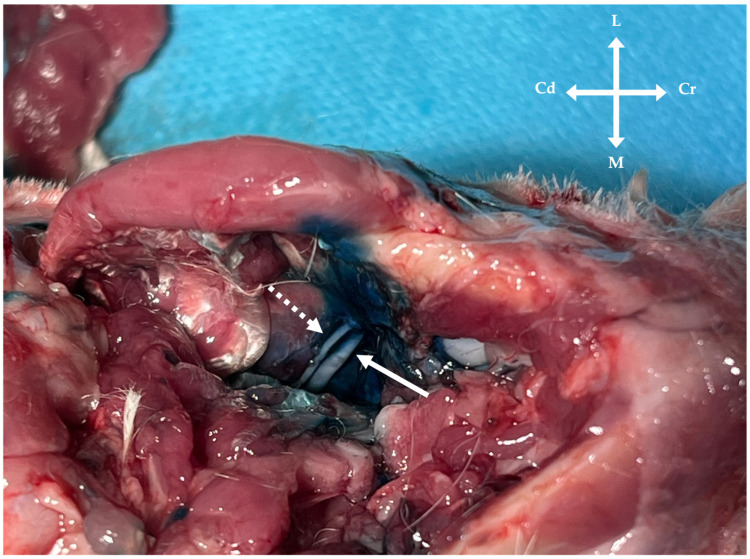
Anatomical dissection of the mandibular region following injection of lidocaine and methylene blue. The mandibular alveolar nerve (solid arrow) is exposed at its entry into the mandibular foramen, showing perineural methylene blue staining. In some specimens, superficial staining was also observed in adjacent tissues, including the lingual nerve (dashed arrow) and surrounding musculature.

No significant differences were observed between the in-plane and out-of-plane approaches for longitudinal staining length (Table 1). Similarly, no statistically significant differences were detected between approaches for ultrasound image quality scores or staining scores (Table 2). Although distribution of the injectate to adjacent nerve branches or muscles was not specifically assessed or quantified, superficial spread of methylene blue to adjacent soft tissues was observed in several specimens, particularly involving the pterygoid musculature and the lingual branch (Figure 8).

## 4. Discussion

The present cadaveric study describes two ultrasound-guided approaches to the mandibular alveolar nerve block in rabbits and provides a comparative evaluation of an in-plane with an out-of-plane technique. The main findings indicate that both approaches allow accurate perineural injectate deposition at the level of the mandibular foramen, with no statistically significant differences in ultrasound image quality or longitudinal dye spread. These results suggest that both techniques are feasible and potentially effective for clinical application in this species.

In the present study, both ultrasound-guided approaches resulted in comparable longitudinal staining lengths and staining scores, supporting their similar effectiveness in targeting the mandibular alveolar nerve. This finding is consistent with reports of other locoregional techniques in veterinary species, in which in-plane and out-of-plane approaches have shown comparable block success rates when the target anatomy is reliably visualised [39,40].

From a technical perspective, the in-plane approach offers the advantage of continuous visualisation of the entire needle shaft and tip during advancement, which is generally considered a safety-enhancing feature of ultrasound-guided regional anaesthesia [41,42]. Continuous needle visualisation allows precise control of needle trajectory and depth, potentially reducing the risk of inadvertent penetration of adjacent structures. However, this theoretical advantage must be balanced against practical limitations, particularly in small species such as rabbits. The use of high-frequency linear transducers, while providing excellent spatial resolution, may limit the available space for needle manipulation due to their footprint, especially in anatomically confined regions such as the intermandibular area [42,43]. In this context, needle insertion during an in-plane approach may be technically more challenging and less ergonomic, as it requires a longer in-plane needle trajectory to reach the target [40]. Moreover, imaging of the mandibular region presents inherent technical challenges. Prominent bony structures may generate acoustic shadowing and restrict the available acoustic window, while adequate visualisation of superficial targets relies on the use of high-frequency linear transducers. Although these probes provide optimal resolution at shallow depths, they may be associated with higher costs, potentially limiting their availability in clinical practice. These factors may have contributed to the continued preference for anatomical or blind techniques in this anatomical region [44,45].

Conversely, the out-of-plane approach may offer greater procedural simplicity and ease of needle insertion, as it requires less lateral space and allows the needle to be introduced closer to the transducer footprint [40,42]. This consideration may become even more relevant when applying the technique described in the present study to rabbits of smaller size than the New Zealand White specimens used here, particularly for the in-plane approach, where needle manipulation may be more difficult. In such cases, the out-of-plane approach may represent a more practical alternative, or the use of smaller-footprint transducers, such as high-frequency intraoperative “hockey-stick” probes, may facilitate the procedure [46].

A critical aspect of mandibular alveolar nerve block techniques is procedural safety. The region of interest for the approaches described in this study contains several neurovascular structures. In particular, attention should be paid to the facial artery and facial vein, which course through the incisura vasorum facialis (mandibular notch), a region located close to the needle insertion point for the out-of-plane approach [47]. Medially, the mandibular salivary gland may also be encountered, while laterally the marginal mandibular branch of the facial nerve runs in close proximity [47,48]. The blind percutaneous technique currently described for mandibular alveolar nerve block in rabbits involves needle insertion medial to the incisura vasorum facialis [15]; therefore, the risk of vascular puncture is also present with blind approaches. However, ultrasound guidance allows real-time identification of surrounding vascular and soft tissue structures and enables adjustment of the needle trajectory on an individual basis, potentially reducing the overall risk of vascular puncture compared with blind techniques, even when needle entry occurs in close proximity to the mandibular notch. Considering that major vascular structures are less frequently encountered at a more cranial level, an ultrasound-guided in-plane approach may mitigate the risk of iatrogenic injury to vascular, glandular, and neural structures, whereas the out-of-plane approach may be associated with a relatively higher risk of such complications. Although procedural complications cannot be assessed in a cadaveric model, these anatomical considerations highlight the importance of careful technique and detailed anatomical knowledge, particularly in live patients. In addition to the risk of iatrogenic injury, blind techniques inherently rely on estimated distances and surface landmarks and therefore carry a risk of inaccurate needle placement and block failure [15]. Ultrasound guidance mitigates these limitations by allowing real-time identification of bony contours and soft tissue interfaces, as well as visual confirmation of injectate spread at the intended target site [21,40].

Another important aspect concerns the functional nature of the mandibular division of the trigeminal nerve. While the mandibular alveolar nerve is primarily sensory, the mandibular nerve also carries motor fibres innervating the muscles of mastication [15,16]. The observation of injectate spread to surrounding muscular structures and lingual nerve suggests that diffusion to adjacent sensory or motor branches cannot be excluded in vivo. This finding is clinically relevant, as unintended spread of local anaesthetic to adjacent branches may result in sensory blockade of the tongue and oral cavity, as well as transient dysfunction of innervated muscles, including the mylohyoid and digastric muscles and intrinsic muscles of the tongue [18]. Although motor effects cannot be evaluated in cadaveric specimens, this consideration is clinically relevant and has been reported in other species following mandibular nerve blocks, potentially leading to self-inflicted lingual trauma in the postoperative period [18]. It is reasonable to assume that a comparable spread of local anaesthetic to adjacent structures may also occur when blind techniques are employed, given that the anatomical target and intended site of injection are similar. Therefore, this phenomenon should be interpreted as an anatomical consideration inherent to the mandibular region rather than a limitation specific to ultrasound-guided techniques.

An additional clinically relevant consideration is the potential alteration of normal anatomy in rabbits affected by advanced dental disease. Mandibular abscesses, osteomyelitis, and severe odontogenic pathology are common in this species and can substantially distort bony landmarks and soft tissue planes [9,12]. In such cases, reliance on standard anatomical references may be unreliable. Ultrasound guidance may partially overcome these challenges by allowing individualised assessment of altered anatomy; however, severe pathological changes may still impair landmark identification. In these scenarios, combining ultrasound guidance with neurostimulation may represent a valuable strategy to further enhance accuracy and safety, as suggested in both human and veterinary regional anaesthesia literature [21,40]. Neurostimulation may be particularly useful in confirming proximity to motor components of the mandibular nerve in live patients, thereby refining needle placement while minimising the risk of intraneural injection or injection at a suboptimal site for effective blockade.

The cadaveric design represents an inherent limitation of this study. Tissue compliance, vascular perfusion, and nerve physiology can differ substantially between cadaveric specimens and live animals, potentially affecting injectate spread and block performance [49]. Consequently, the present results should be interpreted as proof of anatomical feasibility rather than as direct evidence of clinical efficacy. Another limitation of this study is the use of a single injectate volume (0.1 mL/kg) for both approaches. This volume is consistent with recommendations reported by Jekl and Hauptman [15] for blind techniques, in which volumes of 0.15–0.3 mL per rabbit are suggested depending on body size. Future studies should evaluate the clinical efficacy of different volumes and further characterise injectate distribution in cadaveric models. Additionally, although mandibular anatomy is generally conserved across rabbit breeds, pet rabbits—particularly brachycephalic breeds—may exhibit anatomical variations compared with New Zealand White rabbits, potentially necessitating modifications to the described approach. An additional practical consideration relates to hair clipping requirements for ultrasound-guided procedures. Adequate transducer contact and image quality require clipping of the intermandibular region, which may be perceived as undesirable by some owners for cosmetic reasons. In the present study, hair was clipped over the entire head to allow thorough inspection to exclude anatomical abnormalities affecting the mandibular region and the whole head; however, in a clinical setting, clipping would be limited to the intermandibular region only.

Taken together, the findings of this study support the feasibility of ultrasound-guided mandibular alveolar nerve blocks in rabbits using either in-plane or out-of-plane approaches. Both techniques appear capable of achieving consistent perineural injectate distribution at the target site. From a clinical standpoint, approach selection may therefore be guided by operator experience, available equipment, patient anatomy, and procedural ergonomics rather than by inherent differences in efficacy.

Future studies should focus on in vivo clinical validation of this technique, including assessment of analgesic efficacy, duration of blockade, and incidence of complications. The observation of spread to surrounding tissues observed during dissection further supports the need for in vivo evaluation of potential sensory and motor effects associated with this technique. Additionally, comparative studies evaluating ultrasound guidance alone versus combined ultrasound–neurostimulator techniques in rabbits with and without dental disease would provide valuable insights into optimising locoregional analgesia in this challenging species. Finally, further investigation comparing the feasibility and ergonomics of different transducer types, such as standard high-frequency linear probes versus smaller-footprint “hockey-stick” transducers, may help refine the technique and improve its applicability in small patients or anatomically restricted regions.

## 5. Conclusions

This cadaveric study demonstrates the anatomical feasibility of an ultrasound-guided mandibular alveolar nerve block in rabbits using both in-plane and out-of-plane approaches. Both techniques allowed reliable identification of the target region and achieved comparable perineural injectate distribution. Ultrasound guidance may represent a valuable refinement over blind techniques, potentially improving accuracy and procedural control, particularly in challenging anatomical conditions. Further in vivo studies are warranted to evaluate the clinical efficacy, safety, and practical applicability of this technique in pet rabbits undergoing mandibular dental and oromaxillofacial procedures.

## Figures and Tables

**Table 1:** Longitudinal staining length [median (interquartile range)] for the in-plane and out-of-plane approaches for the ultrasound-guided mandibular alveolar nerve block in rabbits.

In-Plane (mm)	Out-of-Plane (mm)	*p* Value
12 (11–15)	11 (9–16)	0.47

**Table 2:** Ultrasound image and staining scores [number (%)] for the in-plane and out-of-plane approaches for the ultrasound-guided mandibular alveolar nerve block in rabbits.

		In-Plane	Out-of-Plane	*p* Value
Ultrasound image quality score	Poor (0)	0 (0%)	0 (0%)	0.15
	Good (1)	3 (25%)	1 (8%)	
	Excellent (2)	9 (75%)	11 (92%)	
Staining score	Poor (0)	0 (0%)	0 (0%)	1.00
	Good (1)	3 (25%)	3 (25%)	
	Excellent (2)	9 (75%)	9 (75%)	

## Data Availability

The original contributions presented in this study are included in the article. Further inquiries can be directed to the corresponding author.
